# Structure of reverse microemulsion-templated metal hexacyanoferrate nanoparticles

**DOI:** 10.1186/1556-276X-7-83

**Published:** 2012-01-20

**Authors:** Alberto Gutiérrez-Becerra, Maximiliano Barcena-Soto, Víctor Soto, Jesús Arellano-Ceja, Norberto Casillas, Sylvain Prévost, Laurence Noirez, Michael Gradzielski, José I Escalante

**Affiliations:** 1 Chemistry Department, University of Guadalajara, Boul. M. García Barragán # 1451, Guadalajara, Jalisco, 44430, Mexico; 2 Stranski-Laboratorium für Physikalische und Theoretische Chemie, Institut für Chemie, Technische Universität Berlin, Straße des 17. Juni 124, Sekr. TC7, Berlin, 10623, Germany; 3 Laboratoire Léon Brillouin (CEA-CNRS), CEA Saclay, Gif-sur-Yvette, 91191, France

**Keywords:** reverse micelles, template method, nanoparticles, nickel hexacyanoferrate

## Abstract

The droplet phase of a reverse microemulsion formed by the surfactant cetyltrimethylammonium ferrocyanide was used as a matrix to synthesize nanoparticles of nickel hexacyanoferrate by adding just a solution of NiCl_2 _to the microemulsion media. Dynamic light scattering and small-angle neutron scattering measurements show that the reverse microemulsion droplets employed have a globular structure, with sizes that depend on water content. Transmission electron microscopy and electron diffraction are used to obtain information about the structure of the synthesized nanoparticles. The results show that the size and shape of the coordination compound nanoparticles correspond with the size and shape of the droplets, suggesting that the presented system constitutes an alternative method of the synthesis of metal hexacyanoferrate nanoparticles.

## Background

Microemulsions represent thermodynamically stable liquid dispersions containing surfactant aggregates, which can often be found in a large region of the phase diagram of two- or multicomponent surfactant systems [1,2]. They exhibit a well-defined structure that is characterized by a typical correlation length in the nanometer scale. Such microemulsions are of special interest because a variety of reactants can be introduced into the nanometer-sized aqueous domains, leading to materials with controlled size and shape [3-9]. Such characteristics play pivotal roles in controlling the physical, chemical, optical, and electronic properties of these nanomaterials. In the past few years, significant research has been conducted in the reverse microemulsion-mediated synthesis of inorganic (metal halides, selenides, or sulfides) and organic (cholesterol, rhodiarome, rhovanil, nimesulide, etc.) nanoparticles [3-13], and there has been substantial progress in the characterization of microemulsions of various types [14].

The synthesis of nanoparticles by reverse microemulsions is viable and attractive because it does not only produce nanoparticles that have a narrow size distribution, but also the particle size can be controlled by varying the microemulsion composition [15]. The reaction in a microemulsion may be conducted in two modes: (1) a multiple microemulsion method, where two or more microemulsions, each containing one reactant, are mixed together [16]. Upon mixing, the droplets collide with one another as a result of the Brownian motion. These collisions lead to the formation of product monomers [7,17,18]. Nucleation takes place in a given droplet when the number of product monomers exceeds the critical nucleation number [19-21]. Further collisions between a droplet carrying a nucleus and another one carrying the product monomers cause the growth of the nucleus [19,22]; (2) in the simple addition type, the reducing or precipitating reagent is directly added to the microemulsion containing the other reactant [23,24], i.e., this mode promotes intramicellar nucleation and growth [22,25]. When particles are formed in single microemulsions, their size and polydispersity are controlled by one or more of the following mechanisms: reaction kinetics, intramicellar nucleation and growth, intermicellar nucleation and growth, and particle aggregation [26,27]. A variation of this synthetic path could proceed by replacing the counterion of the surfactant, and only then the addition of a salt to this reverse microemulsion media. This last method has been successfully used to synthesize nanoparticles using the anionic surfactant AOT [28], for instance, for the case of cobalt ferrocyanide salt nanoparticles [29]. However, to the best of our knowledge, there is no report on the modification of cationic surfactants with ionic coordination compounds such as the cetyltrimethylammonium ferrocyanide [CTAFeII].

Some advantages of this novel cationic surfactant are readily apparent; for instance, inverse microemulsion formed with this surfactant will allow synthesizing different transition metal hexacyanoferrates [Mhcf] by simply adding different salts to the microemulsion media, i.e., with the same surfactant, it is possible to produce different nanoparticles of coordination compounds (M^II^hcf or M^III^hcf). Such compounds and other Prussian blue analogues have been a subject of several studies because of their promising characteristics which include electrochromism, the ability to mediate (electrocatalyze) redox reactions, ionic and electronic (mixed valence electron hopping) conductivities, capability for storage of countercations, and molecular magnetism [30-32].

According to this motivation, in this paper, we studied the formation of a novel type of ferrocyanide-containing cationic surfactant and its ability to form reverse microemulsions. In this work, we use as surfactant a mixture of cetyltrimethylammonium bromide [CTAB] (95 wt.%) and CTAFeII (5 wt.%). The latter was prepared by replacing the bromide (Br^-^) ions of the cationic surfactant CTAB with ferrocyanide ([Fe(CN)_6_]^4-^) ions following a direct metathesis reaction in an aqueous phase [33]. This new surfactant, CTAFeII, presents a very limited area for a microemulsion phase, so when the mixture of surfactants was used, we reach a more extended region of the microemulsion. This can be explained taking into account the interfacial stiffness caused by the bulky molecules of CTAFeII (a huge counterion and four aliphatic chains). However, by adding CTAB molecules, it was possible to obtain an improvement in the interface flexibility. In addition, by changing the surfactant ratio of the mixture, it was found that the system offers better results to the nanoparticle synthesis when a low concentration of CTAFeII (5 wt.%) was used in the surfactant mixture. Furthermore, López-Quintela established that smaller nanoparticles can be obtained in microemulsions when there is a significant difference in the concentrations of the reactants [7].

## Materials and methods

### Materials

All the reactants used in this report were of analytical grade. CTAB was purchased from Sigma-Aldrich Corporation (99%; St. Louis, MO, USA), ferrocyanide salt (K_4_[Fe(CN)_6_]3H_2_O), from J.T. Baker (99%; Deventer, The Netherlands), *n*-hexane (C_6_H_14_) and NiCl_2_6H_2_O, from Caledon Laboratories Ltd. (98% and 99%, respectively; Halton Hills, Canada), *n*-butanol (C_4_H_9_OH), from Productos Químicos Monterrey (99%; Monterrey, Nuevo León, Mexico), and double distilled water, from Selectropura S.A. de C.V. (*σ *= 1.5 to 3 μS/cm; Guadalajara, Jalisco, Mexico). For neutron scattering experiments, D_2_O (99.9% D; Euriso-Top, Gif-sur-Yvette, France) was used instead of H_2_O to increase the contrast and lower the background.

### Synthesis of modified surfactant

The surfactant CTAFeII was prepared by a direct metathesis reaction in an aqueous phase. The detailed procedure is described in the study of Gutierrez-Bercerra et al. [33]. Functional groups of CTAB and CTAFeII were identified by a Fourier transform infrared [FTIR] spectrometer (Spectrum One, PerkinElmer, Waltham, MA, USA). Infrared spectra were recorded in the 400- to 4,000-cm^-1 ^region, with a resolution of 4.00 cm^-1^.

### Phase diagram

The pseudo-ternary phase diagram for the CTAB + CTAFeII + *n*-butanol/*n*-hexane/water system was constructed, considering as surfactant a mixture of CTAB, CTAFeII, and *n*-butanol, using *W *_CTAB_/*W *_CTAFeII _ratios of 0.95:0.05 and the (*W *_CTAB _+ *W *_CTAFeII_)/*W *_but _ratio of 1, where *W *_CTAB_, *W *_CTAFeII_, and *W *_but _are the weights of CTAB, CTAFeII, and *n-*butanol, respectively. A simple titration technique was used to construct the diagram. Microemulsions were prepared by mixing weighed appropriate amounts of the individual components. The amount of *n*-hexane (*W *_hex_) in the surfactant mixture determines the *H *value (*H *= [*W *_CTAB _+ *W *_CTAFeII _+ *W *_but_]/[*W *_hex _+ *W *_CTAB _+ *W *_CTAFeII _+ *W *_but_]), while *W *_w _*= W *_water _*/(W *_water _*+ W *_hex _*+ W *_CTAB _*+ W *_CTAFeII _*+ W *_but_) represents the weight fraction of water used as the titration component. Water was added in small volumes under permanent stirring in a tightly closed vial to avoid evaporation. Then, the vials were placed in a thermostatic bath (25°C) until a homogeneous media is reached.

### Dynamic light scattering

Dynamic light scattering [DLS] measurements were performed using an ALV/CGS-3 goniometer with an ALV/LSE-5004 multiple tau digital correlator (ALV-Laser Vertriebsgesellschaft m-b.H., Langen, Germany). The light source was an He-Ne laser operating at a wavelength of 633 nm. The homodyne intensity autocorrelation function *g *^(2)^(*τ*) was measured at 90°. Data analysis was performed with the normalized intensity autocorrelation function using a third-order cumulant fit [34] that yielded as key parameter the effective collective diffusion coefficient.

### Small-angle neutron scattering

Small-angle neutron scattering [SANS] measurements were done on the instrument PAXY at Laboratoire Léon Brillouin, Gif-sur-Yvette, France. A wavelength of 0.5 nm (FWHM 10%) was selected, and two configurations were used with sample-to-detector distances of 1.25 and 5.05 m.

### Synthesis of nickel hexacyanoferrate

The synthesis of nickel hexacyanoferrate [Nihcf] nanoparticles was carried out at *H *= 0.4 and *W *_w _= 0.09. Appropriated amounts of CTAFeII, CTAB, *n*-butanol, and hexane were mixed until an *H *value of 0.4 was reached, and then it was maintained under stirring. After that, as an aqueous phase, a solution of 5 mM NiCl_2 _was added to the mixture to reach *W *_w _= 0.09. The microemulsion formed was stable for several days and at the same time maintaining a transparent state. Nihcf nanoparticles were separated from the microemulsion media by centrifugation at 9,000 rpm for 10 min. The precipitate was then washed several times with acetone. Despite the washing process, a small quantity of CTAB remained mixed with the nanoparticles. To obtain transmission electron microscopy [TEM] micrographs (JEM-1010, JEOL de Mexico S.A. de C.V., Mexico City, Mexico), a drop of the nanoparticles dispersed in acetone was placed directly on a carbon-coated copper grid. X-ray diffraction [XRD] patterns were recorded with a STOE Theta/theta X-ray diffractometer (STOE & Cie GmbH, Darmstadt, Germany) using a CuKα (*λ *= 0.15406 nm) at room temperature. FTIR spectra of the Nihcf were carried out in a PerkinElmer Spectrum One spectrometer.

## Results and discussion

### Surfactant characterization

**Figure 1:**
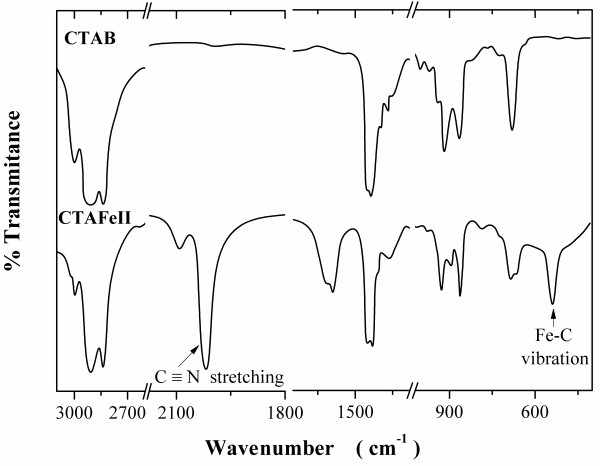
**Surfactant characterization**. Infrared spectra of CTAB (above) and CTAFeII (below).

For comparison, the IR spectra of the surfactants CTAB and CTAFeII are shown in Figure 1. The symmetric (*υ *_s_(CH_2_), *d *^+^) and asymmetric (*υ *_as_(CH_2_), *d *^-^) stretching vibrations of pure CTAB indicate equivalent gauche defects which lie at 2,849 and 2,918 cm^-1 ^[35], as well as those of CTAFeII. The peaks at 3,017 and 1,487 cm^-1^, and at 1,473 and 1,462 cm^-1 ^were attributed to the asymmetric and symmetric C-H scissoring vibrations of CH_3_-N^+ ^moieties and to the CH_2 _scissoring mode, respectively [36]. The above mentioned results indicate that both surfactants possess a long aliphatic chain with a positively charged polar head as expected for the hydrocarbon CTAB structure. On the contrary, two peaks only appear in the CTAFeII: at 595 cm^-1 ^due to the Fe-C vibration and at around 2,000 to 2,100 cm^-1 ^due to the C≡N stretching [37]. Hence, it confirms that indeed the ferrocyanide ion is present in the CTAFeII. The low-spin Fe(II) is diamagnetic and will thus not have electronic transitions. The absorptions near 1,500 and between 1,550 and 1,700 cm^-1 ^can be attributed to overtones and combination tones of OH^- ^and H_2_O fundamental vibrations. The much lower reflectivity of the CTAFeII is a consequence of the high water content, which produces intense absorption with a broad band near 1,550 and 1,700 cm^-1 ^because of the water present. In order to quantify this amount of water in CTAFeII samples, Karl Fisher titrations were carried out, obtaining a 2.8% in weight.

### Phase behavior

**Figure 2:**
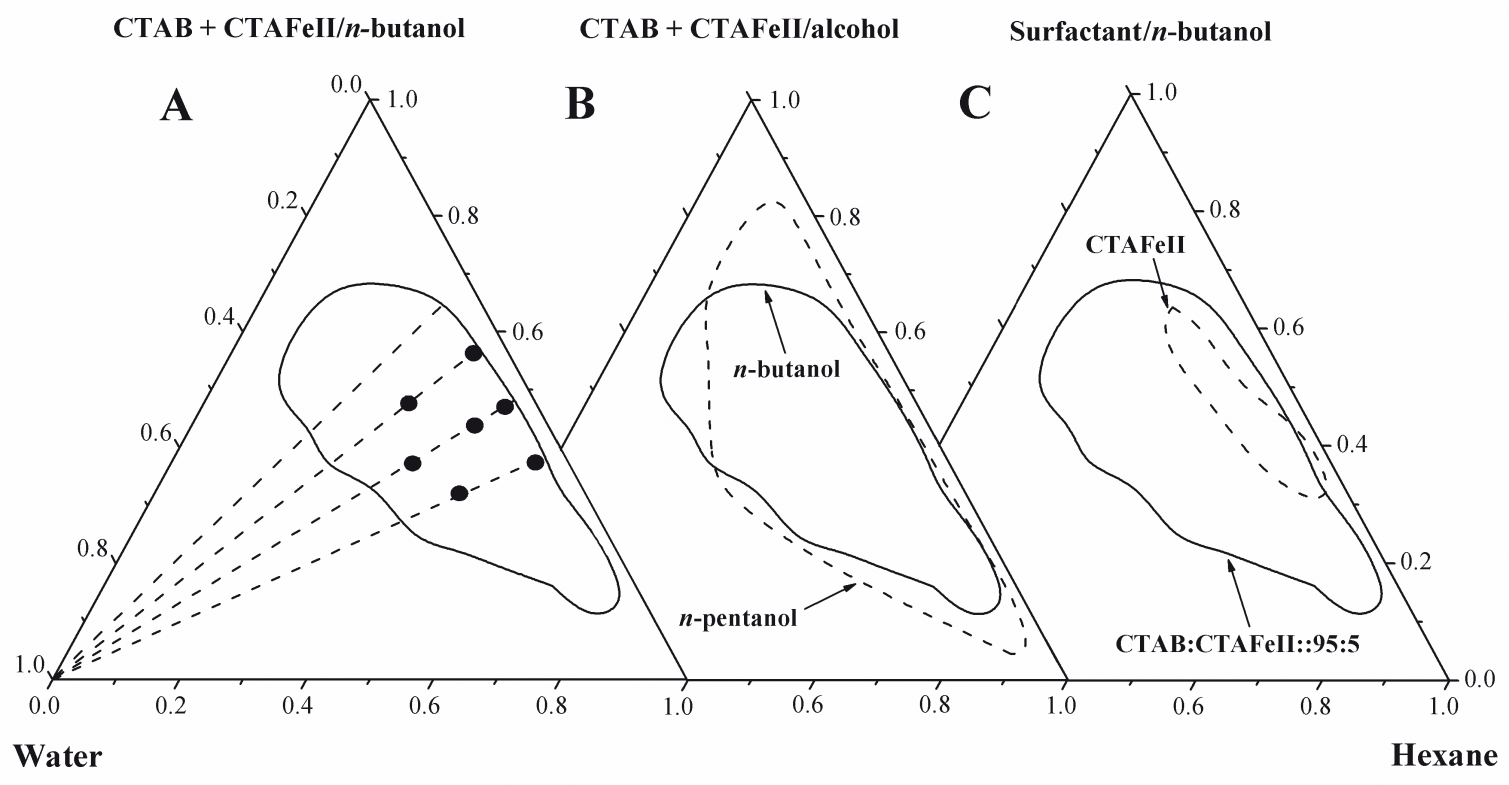
**Phase diagrams**. Pseudo-ternary phase diagrams obtained at 25°C for the systems: (**A**) CTAB + CTAFeII/*n*-butanol/*n*-hexane/water, (**B**) CTAB + CTAFeII/alcohol/*n*-hexane/water, and (**C**) surfactant/*n*-butanol/*n*-hexane/water. The section closed within the solid (or dashed) lines corresponds to the microemulsion phase. The discontinued lines in (A) represent the *H *values used for the measurements. Symbols indicate the compositions for the SANS measurements.

The pseudo-ternary phase diagram obtained for the CTAB + CTAFeII + *n*-butanol/*n*-hexane/water at 25°C is shown in Figure 2A. The boundary between the microemulsion and the non-microemulsion region was established by a systematic titration based on the clear-turbid observation and conductivity measurements (not shown here). This microemulsion region is transparent because of the small dispersion size of water droplets in the system. Outside this area, the mixture is turbid, indicating that the system reaches the solubilization boundary for water and forms big emulsion droplets. In order to study the influence of alcohol, in addition, a phase diagram with *n-*pentanol as cosurfactant was obtained (see Figure 2B). The reverse microemulsion region of both phase diagrams is reached at a similar composition but is more extended for the case of pentanol. Obviously, the effectiveness of the cosurfactant increases with increasing chain length, an effect that is typically observed for the formation of microemulsions induced by the addition of a cosurfactant [38,39]. This indicates that the water solubility has no strong dependence on the type of alcohol and that similar conditions prevail at the amphiphilic interface of the microemulsion aggregates for butanol and pentanol. On the other side, by comparing the phase diagrams in Figure 2C, it is seen that the microemulsion region for the mixture of surfactants examined is much larger than the microemulsion region shown for CTAFeII. This shows that the mixture of surfactants favors larger solubilization of water compared to the pure CTAFeII surfactant.

### Structural characterization

**Figure 3:**
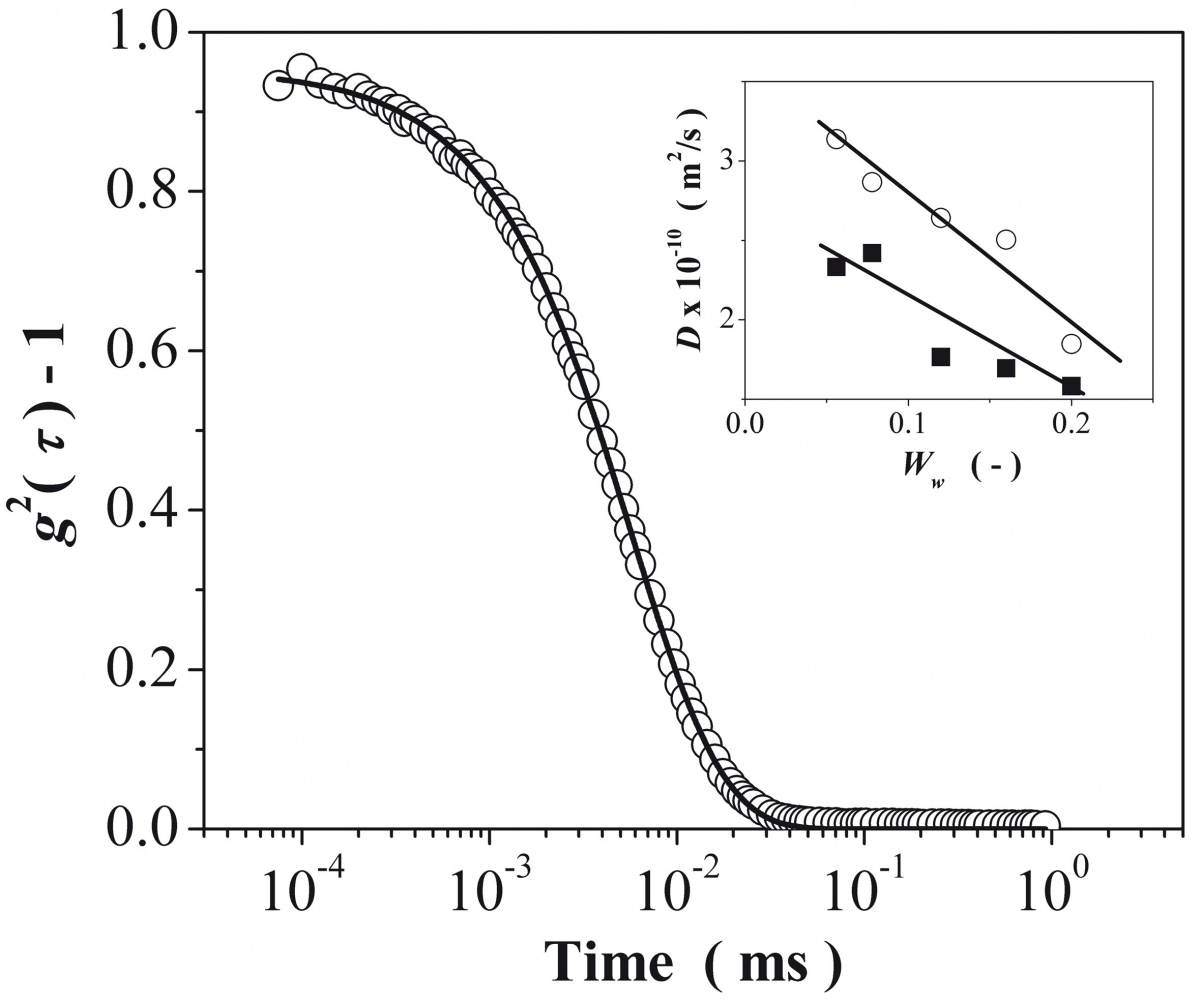
**DLS curve and change of the diffusion coefficient with water content**. Variation of the intensity correlation function *g *^2^(*τ*)-1 with time for the microemulsion structure at *W *_w _= 0.056 and *H *= 0.4. Inset: *D *_eff _vs. *W *_w _for different *H *values, 0.4 (filled square) and 0.5 (empty circle).

Figure 3 shows a representative plot of the correlation function *g *^(2)^(*τ*)-1 obtained for the microemulsions studied. The solid line is a fit to the data using the cumulant method [34]. For a reverse micellar solution, the third-order cumulant expansion of *g *^(2)^(*τ*)-1 varies linearly with *2q^2^τ*. From the slope, the effective collective diffusion coefficients [*D *_eff_] were determined. As a first approximation to determine the droplet size, we considered that the microemulsion is formed by non-interacting droplets. In this condition, the hydrodynamic radius [*R *_h_] can be calculated by the Stokes-Einstein equation *R_h _= KT/6πηD_eff_*, where *k *is the Boltzmann constant, *T*, the temperature, and *η*, the solvent viscosity (the continuous phase in the case of microemulsions). The obtained radii (2.5 to 4.5 nm) are in the same range as those measured by SANS, proving that the non-interacting supposition can be applied in this system without significant error. *D *_eff _and *R *_h _depend on the *H *values (see inset in Figure 3, and Table 1), with larger droplets being present for smaller *H*. An explanation could be that by increasing the hexane content, less butanol is present at the amphiphilic interface (as it becomes dissolved in oil, whereas CTAB and CTAFeII should not be soluble in hexane to any significant extent). Thereby, the total amphiphilic interface available becomes smaller, which then explains the increase in size for a given amount of water. The *R *_h _is also proportional to the relative amount of water contained as demonstrated in earlier works [40,41].

**Table 1:** *D *_eff _and *R *_h _depend on the *H *values

*W *_w_	*H *= 0.4			*H *= 0.5		
	*D *_eff _× 10^-10 ^(m^2^/s)	*R *_h_ (nm)	*p*	*D *_eff _× 10^-10 ^(m^2^/s)	*R *_h_ (nm)	*p*
0.056	2.33	3.15	0.26	3.14	2.37	0.35
0.078	2.42	3.05	0.25	2.87	2.59	0.26
0.120	1.76	4.06	0.31	2.64	2.81	0.28
0.160	1.69	4.22	0.35	2.50	2.96	0.31
0.200	1.58	4.71	0.16	1.85	4.02	0.37

*W *_w_, weight fraction of water; *D *_eff_, effective collective diffusion coefficient; *R *_h_, hydrodynamic radius of the droplets; and *p*, polydispersity obtained by DLS at 25°C for different *H *values.

**Figure 4:**
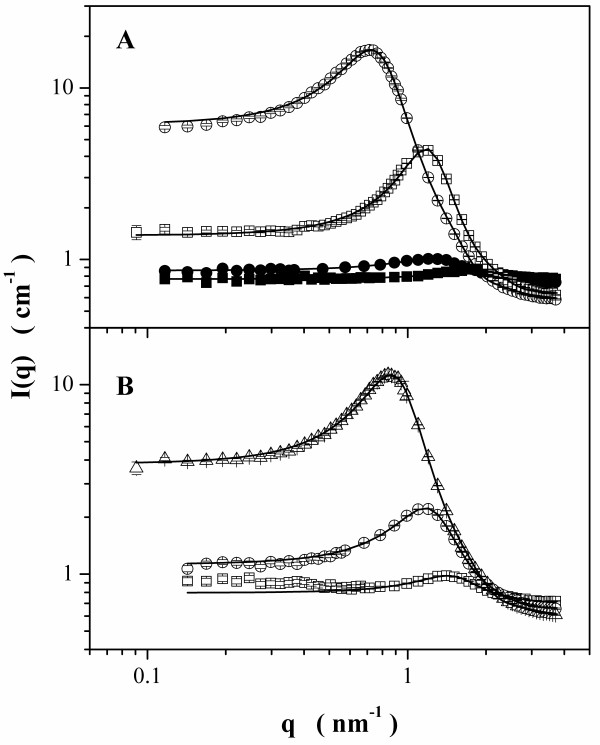
**SANS curves**. SANS spectra (LLB) for reverse micelle solutions of CTAB + CTAFeII/*n*-butanol/*n*-hexane in deuterated water. (**A**) *H *= 0.4 at *W *_w _= 0.05 (filled circle) and 0.20 (empty circle); and *H *= 0.6 at *W *_w _= 0.05 (filled square) and 0.2 (empty square). (**B**) *H *= 0.5 at *W *_w _= 0.05 (empty square), 0.12 (empty circle), and 0.24 (empty triangle). Solid lines are the best fitting obtained by the model (see text).

SANS was applied for obtaining a more comprehensive structural picture in the relevant size range that, for our given *q *range, is about 0.5 to 15 nm, where *q *(*q *= 4 *π*sin (*θ*/2)/*λ*_0_, here, *λ *_0 _and *θ *are the wavelength and the scattering angle, respectively) can be interpreted in terms of distances using *d *= 2 *π */*q*. The obtained scattering curves as a function of *q *are given in Figure 4. At a low amount of water, the spectra have a low intensity. With increasing *W *_w_, the scattering intensity increases and a pronounced angular dependence develop. Apparently, the water core of the aggregates is now large enough to produce a noticeable scattering and is becoming bigger with increasing water content. The shape of the scattering curves at higher *q *already indicates that these reverse microemulsions have a globular structure.

In addition, a correlation peak is visible that becomes much more prominent with increasing water content in the reverse microemulsion, and at the same time, its maximum moves from 1.4 to 0.85 nm^-1 ^(for a fixed *H *of 0.5). This, together with the intensity increase, shows that the aggregates grow substantially in size with increasing water content, where, however, it should be noted that in SANS basically, only the D_2_O core is visible as an aggregate due to the strong contrast between the two isotopes H and D.

The pronounced correlation peak has to be due to steric interactions between the reverse aggregates as electrostatic interactions in the oil-continuous medium should be negligible, but of course, at the concentrations employed, the volume fractions of the amphiphilic material (CTAB, CTAFeII, *n*-butanol) plus D_2_O are in the range of 34% to 59% *v*/*v *and therefore high enough to explain effective repulsion already on the basis of purely steric interactions. In addition, it is well known that in reverse microemulsions, the solvent oil molecules are to a certain extent bound to the reverse microemulsion aggregates [42,43], thereby enhancing the effective volume fraction further.

The first analysis of the SANS data can be performed using the peak position *q *_Peak _to determine the number density ^1 ^*N *of aggregates assuming simple cubic packing: 2*π */*q_peak _*= ^1^*N*^-1/3^. Then, it can be recalled that ^1 ^*N *can be expressed with the volume fraction Φ of the aggregated material and the volume of one aggregate *V*: *N *= Φ/*V*. Assuming spheres, a radius can be deduced. Depending on the choice of material to consider, either only D_2_O or the whole aggregated material (D_2_O, *n*-butanol, surfactant), two values are found that can be considered as values for the core (neglecting dissolved butanol) and for the entire droplet (neglecting the contribution from oil swelling the aliphatic chains of the surfactant); the core radius varies from 0.8 to 3.1 nm and is proportional to the water content of the microemulsion. The difference between the core and the overall radius is in all cases around 1.1 nm (1.03 to 1.25 nm), a reasonable value for the surfactant acting as a shell; taking into account the solvation of this shell by hexane, a higher value would be reached; using Tanford's length, the stretched C15 chain is 2.05 nm; the typically retained value of 75% to 80% of this elongation corresponds to lengths of 1.54 to 1.64 nm; the radius of the tetramethylammonium head group is 0.285 nm; the overall thickness expected for the swollen shell would then be 2.11 to 2.21 nm. However, notice that the *R *_h_, obtained by DLS and SANS, increases roughly linear with the water content as typically observed for reverse microemulsion droplets [44-46].

Two-dimensional data were reduced using BerSANS accounting for dead time, transmission, and background scattering assimilated to the empty cuvette (which means that the incoherent scattering in the spectra still contains contributions from all compounds in the samples including the solvent), and the scattering from H_2_O in a 1-mm cuvette was used to account for the detector pixel efficiency and solid angle variations. Absolute scale was deduced from the evaluation of the direct beam flux. As all corrected scattering patterns are isotropic, they were finally radial-averaged, and data from two configurations were merged.

The whole scattering curves can be described by a model of globular aggregates interacting via an effective hard sphere potential for which the scattering intensity is given by:

(1) $$I\left(q\right){=}^{1}N\cdot {\left({\text{SLD}}_{\text{p}}-{\text{SLD}}_{\text{s}}\right)}^{2}\cdot P\left(q\right)\cdot S\left(q\right),$$

where ^1 ^*N *is the number density of particles, SLD_p _and SLD_s_, the scattering length densities of the particle and the solvent, respectively, *P*(*q*), the particle form factor assuming core-shell spheres, and *S*(*q*), the structure factor accounting for the interparticle interactions, keeping the same density number and using an adjustable hard sphere radius. The core is composed of D_2_O and butanol; the shell of polar charged moieties is composed of D_2_O, butanol, and counterions (bromide is known to adsorb strongly on alkylammonium interfaces, and the amount of ferrocyanide ions is negligible); and the matrix contains hexane and the aliphatic chains of the surfactants (Table 2). The shell thickness was fixed to the dimension of -CH_2_-N(CH_3_)_3 _^+ ^which is 0.57 nm [47]. The incompressibility of all the species was assumed as we do not have access to apparent molecular volumes *in situ*.

**Table 2:** Fit parameters for scattering curves

Material	*v *(Å ^3^)	SLD × 10^-9 ^(cm^-2^)
D_2_O	30.1	63.6
Hexane	218.6	-5.7
Butanol	152.0	-3.30
C_15_H_31 _^-^	432.1	-2.57
CH_2_N(CH_3_)_3 _^+^	110.8	-4.7
Br^-^	51.3	13.2
Fe(CN)_6 _^-^	104.8	100.7

SLD, scattering length densities and *v*, apparent molecular volumes employed in the fitting of the scattering curves.

To evaluate the feasibility of this model, where butanol is absent from the oil phase and partitions between the core and the shell, a comparison of the experimental invariants ${\text{INV}}_{\text{exp}}=\int_{0}^{\infty }\left(I\left(q\right)-I_{\text{inc}}\right)q^{2}dq$ with the theoretical invariants

(2) $$\text{IN}{\text{V}}_{\text{th}}=2\pi^{2}\left[\begin{array}{c} \phi_{\text{oil}}\phi_{\text{shell}}{\left(\text{SL}{\text{D}}_{\text{oil}}-\text{SL}{\text{D}}_{\text{shell}}\right)}^{2}\\ +\phi_{\text{oil}}\phi_{\text{core}}{\left(\text{SL}{\text{D}}_{\text{oil}}-\text{SL}{\text{D}}_{\text{core}}\right)}^{2}\\ +\phi_{\text{core}}\phi_{\text{shell}}{\left(\text{SL}{\text{D}}_{\text{core}}-\text{SL}{\text{D}}_{\text{shell}}\right)}^{2} \end{array}\right]$$

was performed with the partition coefficient of butanol between the core and the shell as the only adjustable parameter; the volume fraction of water in the shell was fixed to be identical to the volume of the tetraalkylammonium group (*ca*. four water molecules per group). Identity was found with the partition of butanol toward the water phase increasing with the amount of D_2_O, except in two cases where the amount of water was too little to actually allow for a core. The maximum volume fraction of butanol in the core does not exceed 16%, only slightly higher than the solubility limit of the alcohol in bulk water (9.5%). Accordingly, we think that our model is reasonable and self-consistent. In all cases, we observe a very good agreement between the values for the droplet core obtained by the analysis of the peak position and by the full fits (Table 3).

**Table 3:** Radius of the microemulsion droplets

*H*	*W *_w_	Volume fraction				*q *_Peak_			Inv	*R *_c _(nm)	(nm)
		Hexane	CTA-X	Butanol	D_2_O	*R *_mic _(nm)	*R *_c _(nm)	*δ* (nm)	*K *_butanol_		
0.4	0.05	0.65	0.14	0.17	0.04	2.29	1.09	1.21	0.07	1.34	2.19
	0.20	0.57	0.12	0.15	0.16	4.34	3.09	1.25	0.14	3.20	3.77
0.5	0.05	0.55	0.18	0.22	0.05	2.19	1.03	1.17	-	1.17	2.00
	0.12	0.52	0.17	0.21	0.09	2.72	1.58	1.14	0.17	1.74	2.35
	0.24	0.47	0.15	0.19	0.19	3.9	2.79	1.12	0.18	2.89	3.28
0.6	0.05	0.46	0.22	0.28	0.04	1.87	0.78	1.09	-	0.90	1.69
	0.20	0.40	0.19	0.24	0.16	2.94	1.91	1.03	0.11	2.04	2.49

*R *_c _and *R *_mic_, the core and the micelle radius, respectively, together with *δ*, shell thickness from the analysis of the peak position; *R *_c_, core radius from the fits; *K *_butanol_, butanol partitioning constant (for the distribution between the core and the shell); *R *_h_, hydrodynamic radius; *W *_w_, weight fraction of water; *q *_Peak_, peak position; Inv, invariant.

### Synthesis of nanoparticles

**Figure 5:**
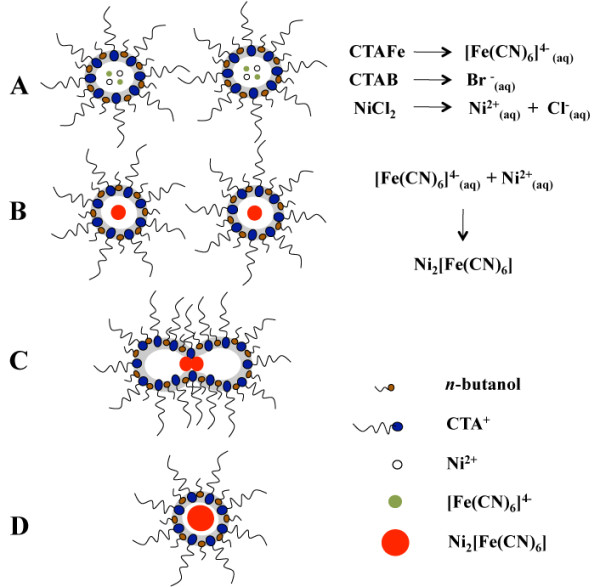
**Mechanisms**. Possible mechanism for the synthesis of Nihcf nanoparticles: (**a**) dissolution of all the ionic species, (**b**) formation reaction of nuclei of Nihcf, and (**c, d**) growth of nanoparticles due to the collisions between the droplets. To simplify, Cl^- ^and Br^- ^ions are not shown.

The formation of Nihcf nanoparticles in this system was carried out in four principal stages as depicted in Figure 5. First, the dissociation of the surfactant counterions (ferrocyanide and bromide) is reached when the solution of NiCl_2 _is added (dropwise under vigorous stirring) to the reverse microemulsion. Then, the nickel and ferrocyanide ions react to form the first nuclei of Nihcf. Once the nuclei are formed, further growth of the particles is taking place via collisions with other microemulsion droplets containing additional salt precursors. Size and shape of nanoparticles are controlled by the steric stabilization provided by adsorbed surfactant molecules on the surface of the nanoparticles [48]. This prospective mechanism still has to be confirmed in more detail by further studies that are currently going on.

**Figure 6:**
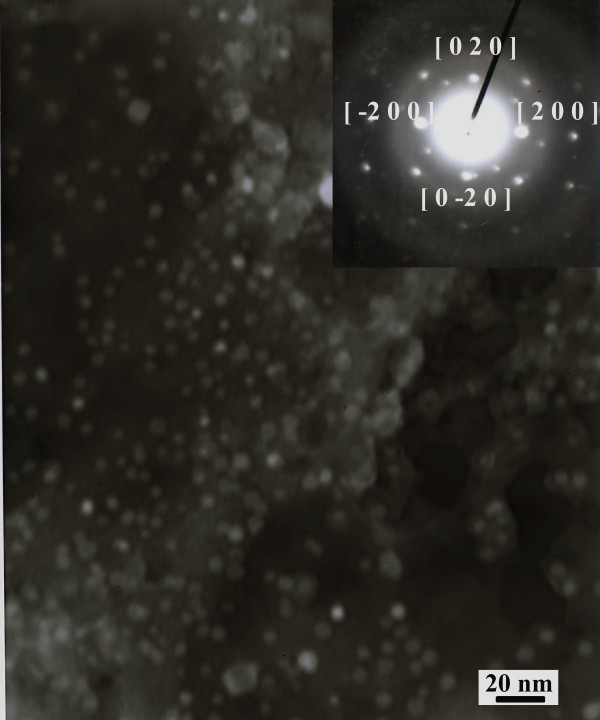
**TEM image of the nanoparticles**. TEM image of the Nihcf nanoparticles synthesized. Inset: electron diffraction.

The reaction between [Fe(CN)_6_]^4- ^ions and the CTAFeII and nickel(II) ions from the aqueous solution produces a colored (yellowish brown) microemulsion without precipitation, suggesting that this reaction is sufficiently facile to allow for the formation of Nihcf, while suppressing at the same time the further growth due to surfactant stabilization of the nanoparticles. Accordingly, the formed particles remain in the nanometer range and are colloidally dispersed (see Figure 6). The inset in this figure shows the indexing of the electron diffraction pattern of the sample with a [40] direction that coincides with the space group F43m characteristic of the Nihcf [49]. Figure 6 confirms the existence of small particles (approximately 6 nm in average size) which have a homogeneous size distribution and correspond to the droplet size of the initial microemulsion droplets (as measured by DLS and SANS).

**Figure 7:**
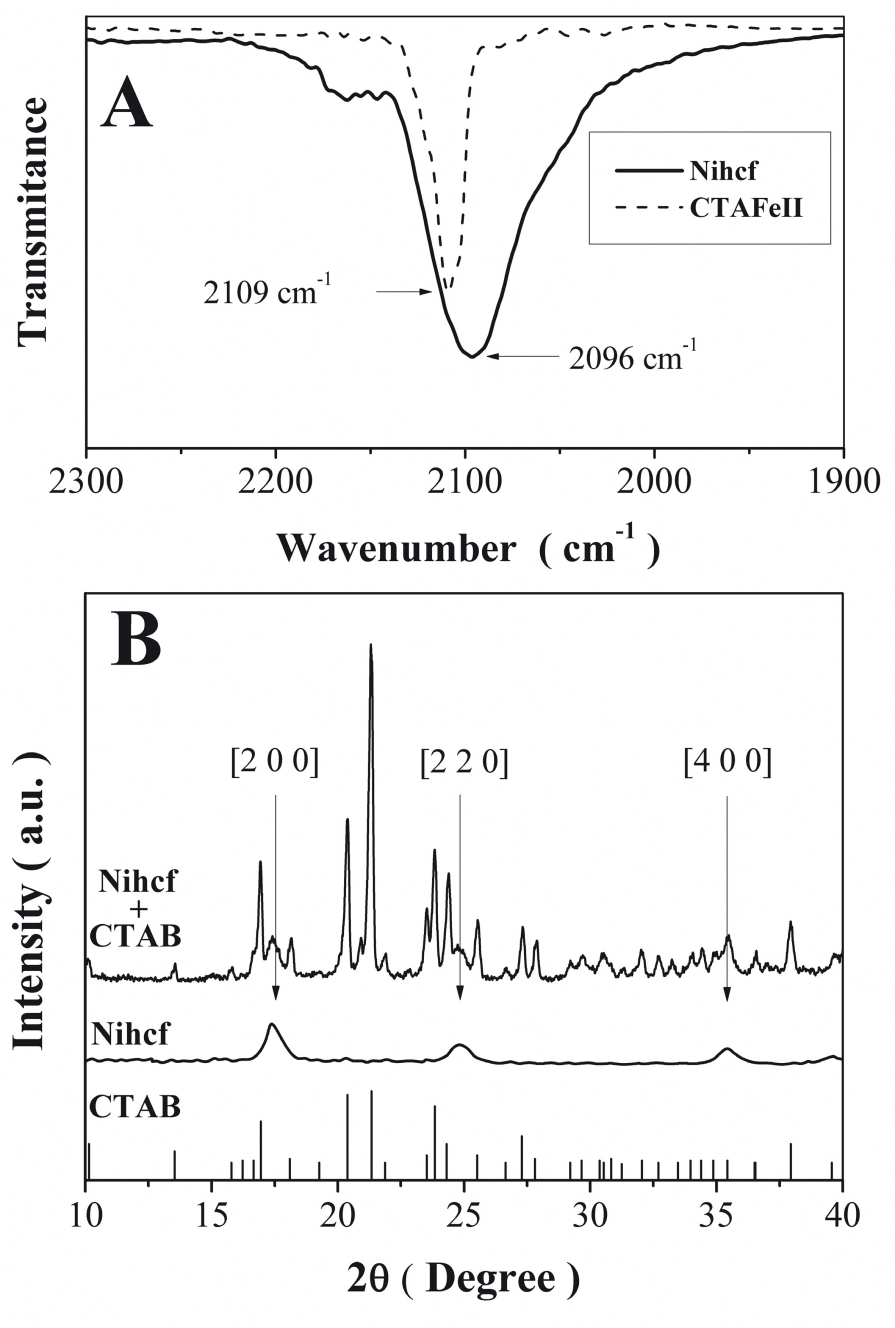
**Nanoparticle characterization**. (**A**) Comparison between the FTIR spectra of the stretching vibration of the cyano group in the Nihcf nanoparticles (solid line) and the surfactant CTAFeII (dashed line). (**B**) XRD patterns of the mixture Nihcf-CTAB; Nihcf nanoparticles are subtracted from the mixture pattern, and the peaks are assigned to CTAB diffraction according to Wong-Ng et al. [51].

XRD and FTIR were performed in order to obtain a better characterization of the Nihcf nanoparticles. Figure 7A shows a comparison between the FTIR spectra of the stretching vibration of the cyano group in the Nihcf nanoparticles (solid line) and the surfactant CTAFeII (dashed line). The absorption band at 2,109 cm^-1 ^can be assigned to the stretching vibration of the C≡N group into the CTAFeII. While for the Nihcf nanoparticles, this absorption band shifts to 2,096 cm^-1 ^and represents the stretching vibration of the cyano group into the cyanoferrate lattice of Nihcf nanoparticles [Ni^2+^-C≡N-Fe^2+^] [50]. On the other hand, Figure 7B shows the XRD pattern of Nihcf nanoparticles mixed with CTAB remained from the washing process. In order to isolate the Nihcf nanoparticle contribution in the diffractogram, the peaks assigned to the diffraction of CTAB [51] were subtracted. The lattice parameter value for the nanoparticles calculated by indexing the peak position using an F43m lattice symmetry is 1.016 nm, close to the 1.000 nm reported elsewhere [49].

An advantage identified in this work for the synthesis of coordination compound nanoparticles is that with this system, different transition Mhcf can be obtained only by varying the transition metal (copper(II), cobalt(II), iron(III), etc.) in the aqueous phase. This constitutes an alternative method using cationic, modified surfactants in reverse microemulsion for the synthesis of this type of nanoparticles.

## Conclusions

In this work, the preparation of nanoparticles of transition Mhcf with a homogeneous size was performed using a simple process in which a droplet is regarded as a nanoreactor. Such soft technique provides good crystallinity in the absence of high temperature and pressure requirements, which favors the formation of small nanoparticles with controlled size and size distribution. Furthermore, it was found that the nanostructure of the particles obtained seems to be related to the structure of the template involved, namely the spherical water pool, at the conditions mentioned in this work.

The ratio of water to surfactant concentration plays an important role in determining the interaction of the water pool with the surfactant or bulk water. Hence, the size of the reverse microemulsion droplets increases as the water pool increases and vice versa. By varying the amount of water content, change in the size of the droplet formed is possible.

Furthermore, using a modified form of the surfactant CTAB (CTAFeII), it was possible to introduce a metal complex ion directly into a reverse microemulsion system without adding a salt as a further component. This procedure allows synthesizing, in a simple way, nanoparticles that correspond in size and shape to the microemulsion droplet morphology. In summary, these experiments demonstrate the feasibility of producing Nihcf nanoparticles using the surfactant CTAFeII.

## Competing interests

The authors declare that they have no competing interests.

## Authors' contributions

AG-B carried out the synthesis and analysis of metal hexacyanoferrate nanoparticles, participated in the sequence alignment, and drafted the manuscript. MB-S participated in the sequence alignment and drafted the manuscript. VS participated in the interpretation and analysis of TEM and diffraction data. JA-C participated in the design of the study. NC helped draft the manuscript. SP carried out the SANS measurements and helped with its analysis. LN carried out the SANS measurements. MG participated in the design and coordination of the study and revised it critically for important intellectual content. JIE conceived the study and participated in the coordination and design of the study. All authors read and approved the final manuscript.
